# Hip complaints differ across age and sex: a population-based reference data for the Hip disability and Osteoarthritis Outcome Score (HOOS)

**DOI:** 10.1186/s12955-018-1022-8

**Published:** 2018-10-11

**Authors:** A Sundén, K Lidengren, E M Roos, L S Lohmander, E Ekvall Hansson

**Affiliations:** 1 Department of Health Sciences, Lund University, Lund, Sweden; 2 Department of Sports Science and Clinical Biomechanics, University of Southern Denmark, Odense, Denmark; 3 Department of Clinical Sciences in Lund, Lund University, Lund, Sweden; 4 Department of Clinical Sciences in Malmö, Lund University, Lund, Sweden

**Keywords:** Osteoarthritis, Hip, HOOS, Reference values

## Abstract

**Background:**

The Hip disability and Osteoarthritis Outcome Score (HOOS) is a self-administered hip-specific questionnaire intended to evaluate symptoms and functional limitations, and it is commonly used to evaluate interventions in individuals with hip dysfunction or hip osteoarthritis. The HOOS consists of 43 questions in five subscales: Pain, Symptoms, Function in daily living, Function in sport and recreation and Hip-Related Quality of Life. This study aimed to establish population-based reference values for the HOOS and to describe the variation of hip-related symptoms in an adult population.

**Methods:**

The HOOS questionnaire was mailed to 840 individuals aged 18–84 years randomly retrieved from a national population record for the Skåne region of Southern Sweden.

**Results:**

The overall response rate was 67%. Older women and men consistently reported more hip-related complaints than those younger. There were significant differences between the oldest and the youngest age groups in all five subscales in women and men.

**Conclusions:**

Hip-related pain, symptoms, activity of daily life and quality of life varied with age and sex in this population-based cohort. Our findings show the importance of using age- and sex-matched reference values for evaluation of outcomes after interventions due to hip-related problems.

## Background

Multi-item disease-specific self-reported outcome measures have been recommended to assess the effect of interventions on hip injury and hip osteoarthritis (OA) [1]. The Hip disability and Osteoarthritis Outcome Score (HOOS) was established as an extension of The Western Ontario and McMaster Universities Arthritis Index (WOMAC) by adding new questions based on over one hundred interviews with individuals with hip disability [2–4]. The HOOS was constructed to better fit, as compared to the WOMAC, patients’ expectations of more demanding physical function – for example for younger individuals and individuals in an early stage of the disease [5]. The HOOS has been recommended specifically for the evaluation of patients with hip OA undergoing non-surgical or surgical interventions such as total hip replacement (THR) [2, 6, 7]. When assessing individuals with hip OA the psychometric properties of the HOOS have shown adequate test-retest reliability, floor and ceiling effects, and construct validity. The HOOS has also been validated for short- and long-term follow-up studies of patients with primary OA assigned for THR [2, 5, 8]. Thus, the HOOS has psychometric properties that enable researchers and clinicians to use it with confidence [5, 9–11].

To facilitate the interpretation of responses to interventions and in clinical decision-making, a comparison with appropriate reference values is essential [12]. However, there is a lack of reference data for many clinical measures from the general population, in particular with regard to the relationship between demographic factors and self-reported hip status [12].

Our study aimed to establish reference values for the HOOS, and to describe the age- and sex-related variation of hip-related pain, other symptoms, functional difficulties during daily life as well as sport and recreation, and hip related quality of life.

## Methods

A population-based sample was randomly chosen from a national population record for the Skåne region (Scania) of Southern Sweden. Everyone in Sweden is registered in the National Population Records, which is updated every six weeks. About 1/9 of the Swedish population is in Skåne – at the time of this study approximately 770,000 individuals aged over 18. Skåne includes both urban and rural communities. A simple sampling method was used. From the national population record we requested a random sample of 60 men and 60 women from each of seven age groups (18–24, 25–34, 35–44, 45–54, 55–64, 65–74 and 75–84), for a total of 840 individuals. These age groups were then collapsed into four age groups (18–34, 35–54, 55–74, and 75–84). This selection of age groups followed the same procedure as an earlier study on reference values for the Knee injury and Osteoarthritis Outcome Score (KOOS) [13]. The number chosen (60 men+ 60 women /10-year stratum) was based on experience from clinical studies using the KOOS, where a clinically significant difference of 10 points has been observed in different populations after interventions [14–16]. No other demographic characteristics besides age and sex were obtained. The HOOS questionnaire was mailed together with a cover letter explaining the purpose of the study, and a prepaid return envelope. The non-responders were reminded twice with the same cover letter as the first one together with a new HOOS questionnaire and a new prepaid return envelope.

### Questionnaire

The HOOS is a self-administered disease-specific questionnaire intended to evaluate symptoms and functional limitations related to hip osteoarthritis. The questionnaire is free of charge and can be downloaded from the KOOS website [17]. The HOOS is available in more than 20 languages; in this study the Swedish version was used. In this study, the Swedish version was used [17]. The HOOS is an adaptation of the KOOS [17–20]. The version used in this study was the HOOS LK1.0 which contains 43 items in five subscales: Pain (Pain) (10 items), Symptoms (Symptoms) (4 items), Function in daily living (ADL) (21 items), Function in sport and recreation (Sport/Rec) (3 items) and Hip-Related Quality of Life (QOL) (5 items). A Likert scale of five response options is used for each item [17].

The HOOS LK1.0 has been revised, and the most recent form the HOOS LK2.0 consists of 40 items assessing the five separate patient-relevant subscales [17]. The HOOS LK2.0 was not available at the time the data of the present study was collected.

### The HOOS scoring

Standardized answer options were given (5 Likert boxes) and each item assigned a score from 0 to 4. The scores from all items within a subscale were then summed. A separate score was calculated for each of the five subscales and then transformed to a 0 to 100 scale where 100 represented the best result. Traditionally in orthopedics, a score of 100 indicates no problems and 0 indicates extreme problems. The normalized score is transformed to meet this standard [17, 19]. In the present study, if the number of missing items was more than 50% in a subscale, the response was considered invalid and no subscale score was calculated in accordance with the HOOS 2013 scoring manual [17].

### Statistical analysis

The statistical analyses were performed with SPSS 21.0 for Windows (SPSS Inc., Chicago, Il, USA). To increase power, compensate for non-responders, and minimize the number of comparisons made, the original seven age groups were collapsed into four age groups when testing for differences due to age and sex. Reference values for the KOOS were similarly reported [13]. Analysis of variance, ANOVA, was used to examine differences between groups. A post hoc test with Bonferroni correction was used to protect from Type 1 Error due to multiple comparisons. Some of the data were not normally distributed, but the sample size in each group was large enough (*n* ≥ 30) to apply the central limit theorem, which gives normally distributed sample means [21].

## Results

**Fig. 1:**
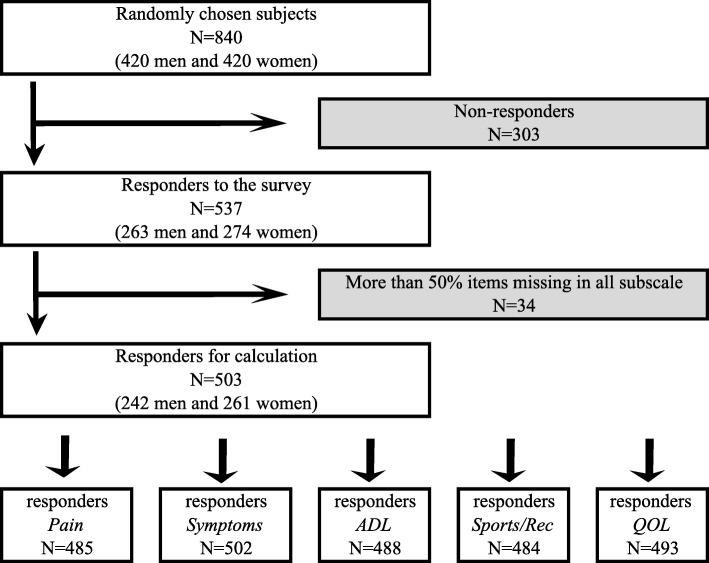
Flow chart detailing the study procedure and formation of the patient cohort.

Eight hundred and forty questionnaires were sent out, of which 45 were returned undelivered because the intended recipient was deceased or had moved to an unknown address (Fig. 1). Of the remaining 795 questionnaires, 537 were returned (response rate 67%). In 34 cases, more than 50% of items were missing for all subscales and no scores could be calculated. Scores for at least one subscale could thus be calculated in 503 subjects (63%; 242 men and 261 women) (Fig. 1). The highest response rate, 73%, was found in the age groups 55–74 and 75–84.

**Fig. 2:**
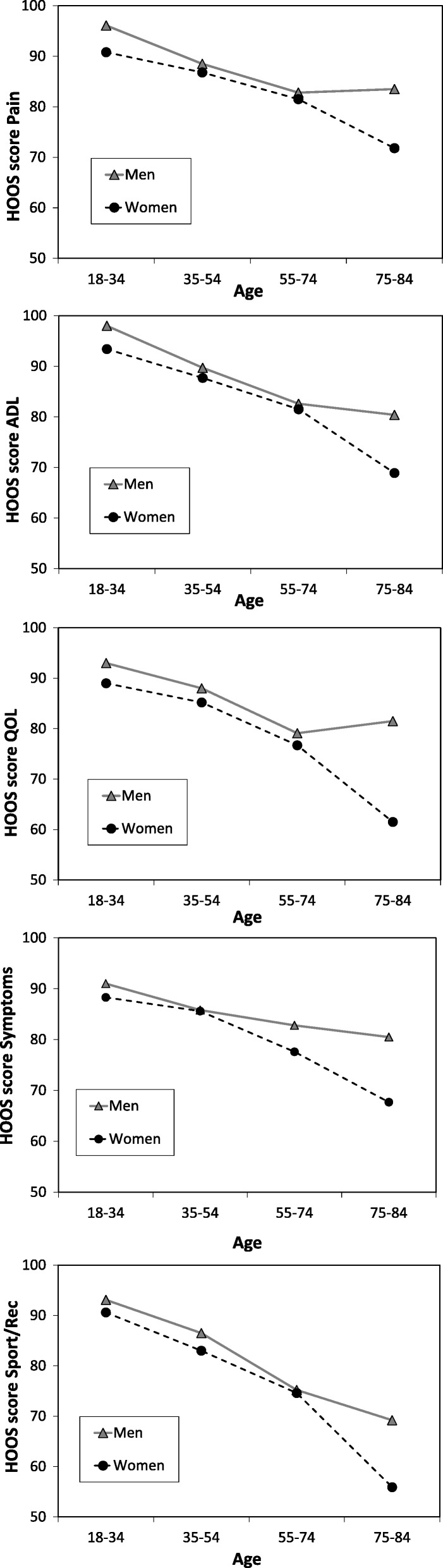
Mean HOOS scores of the subscales Pain, Symptoms, ADL, Sports/Rec and QOL for men and women in different age groups.

Age-related differences were studied separately in men and women in the four age-collapsed groups (Fig. 2). For both men and women, hip function decreased with increasing age.

### Age-related differences in women

The oldest age group (75–84 years) reported statistically significantly more disability than the youngest group (18–34 years) in all five subscales: Pain (72 vs. 91, *p* < 0.001), Symptoms (68 vs. 88, *p* < 0.001), ADL (69 vs. 93, *p* < 0.001), Sport/Rec (56 vs. 91, *p* < 0.001) and QOL (61 vs. 89, *p* < 0.001).

Significant differences were also found in the subscales ADL (69 vs. 81, *p* = 0.043), Sport/Rec (56 vs. 75, *p* = 0.017) and QOL (61 vs. 77, *p* = 0.028) between women in the two oldest age groups (75–84 years and 55–74 years) with more complaints in the oldest age group.

Significant differences were found between the oldest age group (75–84 years) and the age group 35–54 years in Pain (72 vs. 87, *p* = 0.007), Symptoms (68 vs. 86, *p* = 0.001), ADL (69 vs. 88, *p* < 0.001), Sport/Rec (56 vs. 83, *p* < 0.001) and QOL (62 vs. 85, *p* < 0.001) with more complaints in the oldest age group.

Between the age group 55–74 years and the youngest age group (18–34 years) significant differences were found in Symptoms (78 vs. 88, *p* = 0.026), ADL (82 vs. 93, *p* = 0.012), Sport/Rec (75 vs. 91, *p* = 0.011) and QOL (77 vs. 89, *p* = 0.031) with more complaints in the age group 55–74 years.

No significant differences were found between the age group 35–54 years and the youngest age group (18–34 years). (Table 1 and Fig. 2).

**Table 1:** Age-specific HOOS scores given as mean, standard deviation (SD), 95% confidence interval (CI) of the mean, and median, for woman (W)) and men (M) in the different age groups

Age group	18–34	35–54	55–74	75–84
Pain				
Wn	63	76	80	35
MeanSD	90.8 ± 15	86.8 ± 21.7	81.5 ± 23.9	71.8 ± 29.8
95%CL	87.1–94.5	81.9–91.7	76.3–86.7	61.9–81.6
Median	97.5	100	94.7	82.5
Mn	46	73	74	38
MeanSD	96.1 ± 9.9	88.5 ± 19.9	82.8 ± 22.6	83.5 ± 24.3
95%CL	93.2–98.9	83.9–93.1	77.7–88	75.8–91.3
Median	100	100	92.5	98.8
Symptoms				
Wn	65	78	82	35
MeanSD	88.3 ± 16.3	85.6 ± 20.9	77.6 ± 24.4	67.7 ± 29.6
95%CL	84.3–92.2	80.9–90.2	72.3–82.9	57.9–77.5
Median	93.8	96.9	87.5	77
Mn	50	73	78	41
MeanSD	91 ± 14	85.8 ± 20.2	82.8 ± 23.7	80.5 ± 22.2
95%CL	87.6–95.1	81.2–90.4	77.5–88	73.8–87.3
Median	100	100	93.8	87.5
ADL				
Wn	64	77	80	34
MeanSD	93.4 ± 14.6	87.7 ± 22.2	81.5 ± 24	68.9 ± 32
95%CL	89.8–97	82.8–92.7	76.3–86.6	58.1–79.6
Median	100	100	95.8	75
Mn	50	71	75	38
MeanSD	98 ± 7.4	98.7 ± 19.5	82.6 ± 24.4	80.4 ± 28.7
95%CL	95.7–99.8	85.2–94.2	77.1–88.1	71.2–89.5
Median	100	100	95.2	98.8
Sport/Rec				
Wn	63	77	77	34
MeanSD	90.6 ± 19.4	83 ± 29.3	74.6 ± 32.3	55.9 ± 39.4
95%CL	85.8–95.4	76.5–89.6	76.4–81.8	42.5–69.4
Median	100	100	91.7	58.3
Mn	48	72	75	39
MeanSD	93.1 ± 20.7	86.5 ± 23	75.2 ± 33.5	69.2 ± 38.2
95%CL	87.2–98.9	81.2–91.8	62.7–82.8	57.2–81.2
Median	100	100	91.7	91.7
QOL				
WN	64	77	82	34
MeanSD	89 ± 17.4	85.2 ± 23.7	76.7 ± 28.3	61.5 ± 36.9
95%CL	84.7–93.2	79.9–90.5	70.6–82.9	49.1–73.9
Median	100	100	90	67.5
Mn	48	73	76	39
MeanSD	93 ± 13	88 ± 20	79.1 ± 27.4	81.5 ± 25.7
95%CL	89–96.5	83.4–92.6	72.9–85.2	73.5–89.6
Median	100	100	93.8	100

### Age-related differences in men

Significant differences were found between the oldest age group (75–84 years) and the youngest age group (18–34 years) with more hip problems in the oldest age group in four subscales: Pain (83 vs. 96, p = 0.03), ADL (80 vs. 98, *p* = 0.001), Sport/Rec (69 vs. 93, *p* < 0.001) and QOL (81 vs. 93, *p* = 0.036).

Significant differences were also found between the age groups 75–84 years and the second youngest age group (35–54 years) in Sport/Rec (69 vs. 86, *p* = 0.016), with more complaints in the oldest age group.

Between the second oldest age group (55–74 years) and youngest age group (18–34 years) significant differences were found in Pain (82 vs. 96, *p* = 0.002), ADL (83 vs. 98, p < 0.001), Sport/Rec (75 vs. 93, *p* = 0.001) and QOL (79 vs. 93, p = 0.001) with more hip problems in the age group 55–74 years.

No statistically significant differences were found in any of the five subscales between the age group 75–84 and the age group 55–74 years, age groups 55–74 years and 35–54 years, or between the two youngest age groups (35–54 years and 18–34 years). (Table 1 and Fig. 2).

### Sex-related differences

In the oldest age group, statistically significant differences between the sexes were found for.

Symptoms (68 vs. 80, *p* = 0.019) and QOL (61 vs. 81, *p* = 0.001) where women had more hip problems than men. In the youngest age group, women had statistically significantly more hip problems than men in Pain (91 vs. 96, *p* = 0.003), ADL (93 vs. 98, *p* = 0.002) and in QOL (89 vs. 93, *p* = 0.004).

No significant differences were found between the sexes in the age groups 55–74 years and 35–54 years. (Table 1 and Fig. 2).

## Discussion

The main aim of this study was to establish reference data for the HOOS, a patient-reported outcome instrument for individuals with hip OA. To our best knowledge, this study is the first to report population-based reference data for the HOOS.

The difference in reported hip complaints between men and women was most pronounced in the oldest age group (75–84 years), with women reporting markedly more complaints than men, especially in QOL, where the difference was 20 points (81.5 vs. 61.5 points). In Pain, Symptoms, ADL and Sports/Rec the difference was 11.5–13.3 points also indicating more complaints in women. In the youngest age group, women also reported more hip complaints than men in Pain, Symptoms and ADL.

These results differ from those in the KOOS study (a similar study population randomly chosen from the same national population in Skåne), where men aged 75–84 reported more complaints than both younger men and age-matched women for all subscales but Sport/Rec and women aged 75–84 reported fewer complaints than those aged 55–74 [13]. In agreement with the present study the prevalence of joint complaints was reported higher in women than in men and increases with age [22–24].

The differences between the age groups in women was most pronounced in QOL where the difference was 15.2 points (76.7 vs. 61.5) and in ADL 12.7 points (81.5 vs. 68.8) between the oldest 75–84 and the second oldest 55–74 age group with older women reporting more complaints.

The differences between the age groups in men were most pronounced in Sport/Rec where the difference was 12.3 points (86.5 vs. 74.2) between the age groups 35–54 and 55–74.

Similar to other population-based study cohorts, this group of individuals randomly selected from a general population certainly included individuals with osteoarthritis, other musculoskeletal disorders and comorbidities influencing their response to the HOOS questionnaire. It is known that musculoskeletal disorders affects women more commonly than men, and the prevalence increases with age [22].

We collected data from a wide age range and could therefore study the hip-related self-reported complaints across an adult population. However, although an acceptable response rate was acquired, we cannot disregard the fact that 33% did not answer the questionnaire, and for whom we do not know the reasons. With missing values, there is always the possibility that the participants who declined to respond were too healthy or too sick to be interested, which would mean that the HOOS scores presented in this study could be an under- or over-estimation of the true size of the problem.

Despite the differences in item content in subscales between the HOOS LK1.0 and 2.0, the age- and sex-specific values in the population of this study should be applicable to both versions of the HOOS [17]. Five questions in the HOOS LK1.0 are not included in the HOOS LK2.0, but the content in these removed questions is included in other items in the HOOS LK2.0. Three items were moved from one subscale in the HOOS LK1.0 to another subscale in the HOOS LK2.0. The HOOS subscales scores are not dependent on the number of items in the respective subscales since the total score of each subscale is divided by the possible maximum score for the subscale [17].

## Conclusion

Hip-related pain, symptoms, function of daily life and quality of life varied with age and sex in a population-based sample aged 18–84 years. These findings highlight the importance of using age- and sex-matched reference values in studies evaluating individuals with hip complaints.

## Abbreviations

ADL: Activity limitations-daily living
OA: Osteoarthritis
QOL: Hip-Related Quality of Life
Sport/Rec: Sport and Recreation Function
the HOOS: The Hip disability and Osteoarthritis Outcome Score
the KOOS: The Knee injury and Osteoarthritis Outcome Score
THR: Total hip replacement
WOMAC: The Western Ontario and McMaster Universities Arthritis Index
